# Comparative Analysis of Selective Mining and XRT Sensor-Based Sorting for Copper Ore Pre-Concentration: Preliminary Studies Assessing Method Potential

**DOI:** 10.3390/s26010261

**Published:** 2026-01-01

**Authors:** Jakub Progorowicz, Jakub Kurty, Michal Marcin, Martin Sisol, Anna Romańska

**Affiliations:** 1 Institute of Earth Resources, Faculty of Mining, Ecology, Process Control and Geotechnologies, Technical University of Košice, Letná 1/9, 042 00 Košice, Slovakia; jakub.p@comex-group.com (J.P.); jakub.kurty@tuke.sk (J.K.); michal.marcin@tuke.sk (M.M.); martin.sisol@tuke.sk (M.S.); 2 Comex Polska Sp. z o. o., ul. Kamieńskiego 51, 30-644 Kraków, Poland; 3 Faculty of Electrical and Computer Engineering, Cracow University of Technology, Warszawska 24, 31-155 Cracow, Poland

**Keywords:** sensor-based sorting, X-ray transmission, XRT, pre-concentration, preconcentration, selective mining

## Abstract

This study evaluates sensor-based pre-concentration using XRT technology as an alternative to selective mining for low-grade European copper ores (0.48% Cu), addressing the need for sustainable beneficiation amid declining ore grades and environmental pressures in green mining initiatives. Copper ore samples from Złote Hory mine (Czech Republic) were selectively extracted, mixed (1:1:1 ore 8–16 mm/ore 16–32 mm/waste rock 8–32 mm), and analyzed on Comex’s LSX-MAX laboratory sorter with dual-energy XRT sensors, calibrated for maximum product recovery via density-based classification into High-Density (product) and Low-Density (waste) fractions. Sorting achieved a 1:1 product-to-waste mass split from feed (Cu = 0.5%, 100% mass), yielding pre-concentrate at 0.91% Cu (52.08% mass yield, 95.67% recovery) and waste at 0.04% Cu (47.92% mass, 4.33% loss)—a 1.82x grade upgrade superior to mixed feed and 1.42x superior to selective mining (0.64% Cu at 66.21% yield). Combined approaches promise further optimization; future work will assess downstream grinding/flotation impacts, industrial scaling, and economic/environmental benefits.

## 1. Introduction

This publication is the first in a planned series based on research conducted under the project titled “MineCuON—Mining pre-concentration system for copper ores based on XRT and XRF sensors” [1], implemented as a part of the EIT Raw Materials program EIR Regional Innovation Scheme (RIS) [2]. The project focuses on the application of various sensor technologies—specifically “X-ray Transmission (XRT)”, “X-ray Fluorescence (XRF)”, and additionally optical “Short-Wave Infrared (SWIR)” cameras operating up to 2500 nm—to study copper ore samples sourced from different European mines at various stages of the mineral processing stream.

The application of complex sensor configurations, primarily involving X-ray Transmission (XRT), X-ray Fluorescence (XRF), and Short-Wave Infrared (SWIR) cameras integrated with artificial intelligence (AI) systems, significantly enhances the effectiveness of ore structure mapping and the differentiation of valuable components from waste material. This innovative approach not only improves the quality of the pre-concentrate obtained but also minimizes material losses and reduces the environmental impact of the process. Consequently, the project contributes substantial added value to the field of green mining technologies and Industry 4.0 solutions within the mining sector [3,4,5].

The effective pre-concentration of low-grade, highly disseminated copper ores enabled by this technology increases the content of the valuable component in the stream directed toward further beneficiation stages. This allows for a reduction in the volume of ore processed, subsequently lowering operational costs, energy consumption, and CO_2_ emissions. Moreover, it proportionally decreases the volume of material transported, crushed, milled, screened, and floated, which directly reduces electricity demand and wear on machinery and equipment. This leads to cost savings, increased revenues, and a reduction in CO_2_ emissions, alongside the decreased consumption of flotation reagents and the quantity of fine flotation tailings [6,7].

Studies by independent research teams on other raw materials, such as high-sulfur coal [8], demonstrate the high sensitivity of grinding and flotation processes to feed quality. This quality can be enhanced through pre-concentration using sensor-based sorting. Concurrently, the authors are investigating the beneficiation of non-copper raw materials and preparing a comprehensive publication on coal sorting analysis. The primary objective of this research is to increase the calorific value of the feed while significantly reducing the ash and sulfur content.

The current research field involves ongoing debates regarding the optimal integration of these sensor technologies and AI-driven data fusion, particularly concerning their impact on downstream mineral processing stages and environmental sustainability. Some studies emphasize the superior selectivity and resolution of XRT for dense mineral phases, while others highlight the complementary capabilities of XRF or SWIR in surface mineral identification. The synergistic use of multi-sensor data presents both challenges and opportunities for achieving greater sorting precision and process efficiency [9,10].

The main aim of this work is to evaluate the performance and integration of XRT, XRF, and SWIR sensor technologies coupled with AI data processing in the pre-concentration of copper ores, assessing their impact on process optimization, cost reduction, and environmental footprint. The principal conclusions demonstrate that such integrated sensor-based sorting systems can revolutionize mining operations by promoting more sustainable and economically viable beneficiation pathways.

This article addresses the comparison between the currently implemented selective mining method and preliminary studies on sorting using XRT sensors on copper ore from the underground Zlote Hory mine operated by DIAMO, Stráž pod Ralskem in the Czech Republic [11].

Selective mining in this context refers to a method where before extraction, it is determined whether a given rock mass should be classified as waste rock or as ore based on its content of the valuable component. This approach allows for the targeted exploitation of mineralized zones, reducing the amount of waste material processed and improving overall resource efficiency. By selectively extracting only ore-bearing rock, operational efficiency is enhanced, and the unnecessary handling of barren material is minimized, which results in economic and environmental benefits [12].

## 2. Materials and Methods

### 2.1. Sample Material

Selectively extracted material from the Zlote Hory DIAMO mine in the Czech Republic was delivered in three forms:

- Ore, grain size of 8–16 mm.
- Ore, grain size of 16–32 mm.
- Waste rock, grain size of 8–32 mm.

Each sample was divided using a Jones divider to prepare control samples for further testing and sampled for chemical analysis to determine the copper content in each sample. For research purposes, to achieve controlled feed quality, the delivered materials were mixed in equal proportions (1:1:1) to create a feed sample. After mixing, the material was representatively divided using a Jones divider into samples for sorting technology testing and for chemical analysis to determine the actual copper content in the mixed feed. The feed was then analyzed using XRT technology. This analysis enabled classification and sorting for pre-concentration.

### 2.2. XRT Analysis

The X-ray Transmission (XRT) analysis system employs a sensor array arranged in a linear “sandwich-type” configuration. This setup includes two detectors positioned sequentially along the X-ray beam path. The detector closest to the X-ray source is designed to register low-energy photons. Behind this, separated by a copper filter, is a second detector responsible for capturing high-energy photons [13].

**Figure 1:**
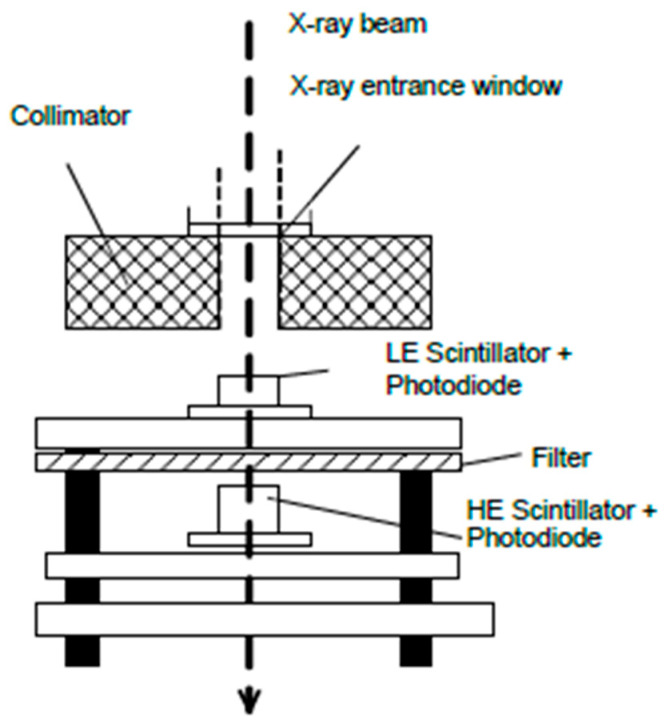
Concept drawing of the dual energy detection [14].

As X-ray radiation passes through the object under analysis, the degree of absorption varies with the material’s composition and density. Due to this differential absorption, the two detectors receive distinct intensity levels of X-ray photons (Figure 1) [14]. These signals are digitized and undergo numerical combination processing [15] or are processed using artificial intelligence algorithms [16]. The resulting data provide an interpretation of the atomic density of the analyzed material, facilitating accurate material differentiation. By applying appropriate image processing methods, dual energy XRT analysis compensates for differences in particle size (with an adopted size ratio limit of 3), and therefore the simultaneous use of a 3D camera to measure the shape of individual particles is not required.

**Figure 2:**
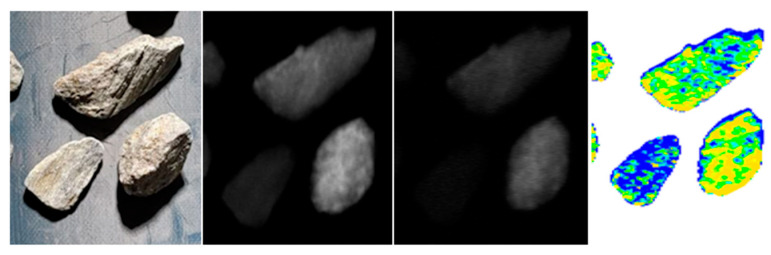
From left to right: photograph of scanned objects; raw XRT_LE (low-energy) image scan; raw XRT_HE (high-energy) image scan; and interpretation resulting from numerical combination of XRT_LE and XRT_HE images.

**Figure 3:**
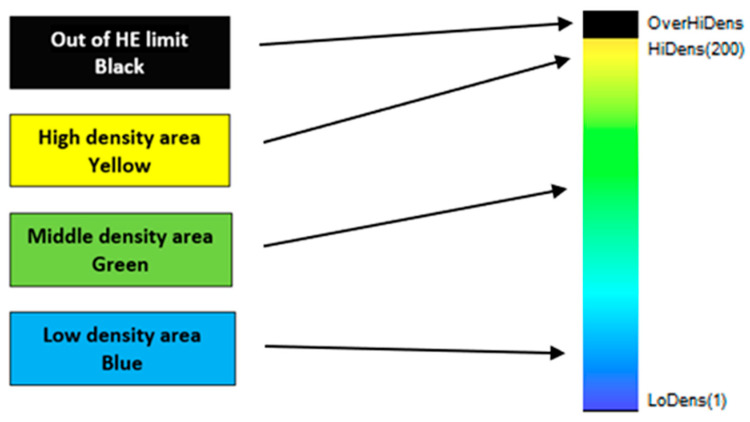
Description color scale related to relative density—greater color variation between individual rock samples increases the likelihood of achieving better separation quality.

This dual-detector approach allows for enhanced material discrimination by leveraging differences in X-ray photon energy absorption, improving sorting accuracy in mineral processing and related applications (Figure 2 and Figure 3). 

### 2.3. Chemical Analysis

Samples were pulverized to achieve a grain size with 85% passing below 0.075 mm. The resulting samples were analyzed using accredited analytical protocols specific to each type. Quantitative elemental analysis was performed using X-ray fluorescence spectrometry (XRF) (SPECTRO Analytical Instruments GmbH, Kleve, Germany).

### 2.4. Laboratory Equipment

The research was conducted using the modular laboratory analysis and sorting unit LSX-MAX, designed and manufactured by Comex Polska sp. z o. o., Kraków, Poland [17]. The device features ergonomic dimensions and ensures beam geometry consistency across the different analytical technologies it employs, akin to the full-scale industrial sorting systems. The conveyor belt speed in the laboratory systems is approximately 1.5 m/s, with image resolution corresponding to a full production speed of 3.0 m/s. The final feeding conveyor section acts as a reject chute, allowing the scanned material to be separated into two products based on classification using sensors. It is equipped with imaging sensors based on multiple technologies, including XRT, XRF, Gamma, UV, VIS-NIR, SWIR, and 3D. This multimodal setup enables fusion imaging and multiparametric data matrix analysis.

**Figure 4:**
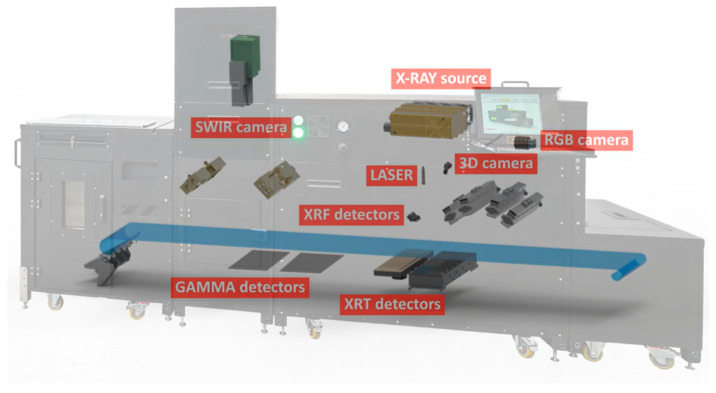
Visualization of the imaging technology layout in the LSX-MAX laboratory system by Comex Polska sp. z o. o.

In this particular study, the analysis was confined to XRT technology. However, future iterations of the research plan to expand the array of analytical technologies to include both XRF and SWIR for a more comprehensive evaluation (Figure 4).

For the analysis, an X-ray tube operating at 160 kV and 2.5 mA (400 W) was employed, together with X-ray sensors featuring a pixel pitch of 0.8 mm.

## 3. Results

### 3.1. Feed

In reference to the sample preparation procedure described in Section 2.1, the samples provided for the chemical analysis of individual portions of the feed and the mixed feed yielded the following results.

The average copper content in copper ore at the level of 0.48% is a value that well reflects European copper deposits and provides a good starting point for research in light of their broader application [18]. It is worth noting the high effectiveness of selective mining given the quality of waste rock (Table 1, Table 2, Table 3 and Table 4), which contains no copper at all.

### 3.2. XRT Mapping

A representative sample separated from the feed was scanned to determine the separation threshold. Analysis parameters were set to maximize product recovery (a situation where product is lost in waste is unfavorable).

**Figure 5:**
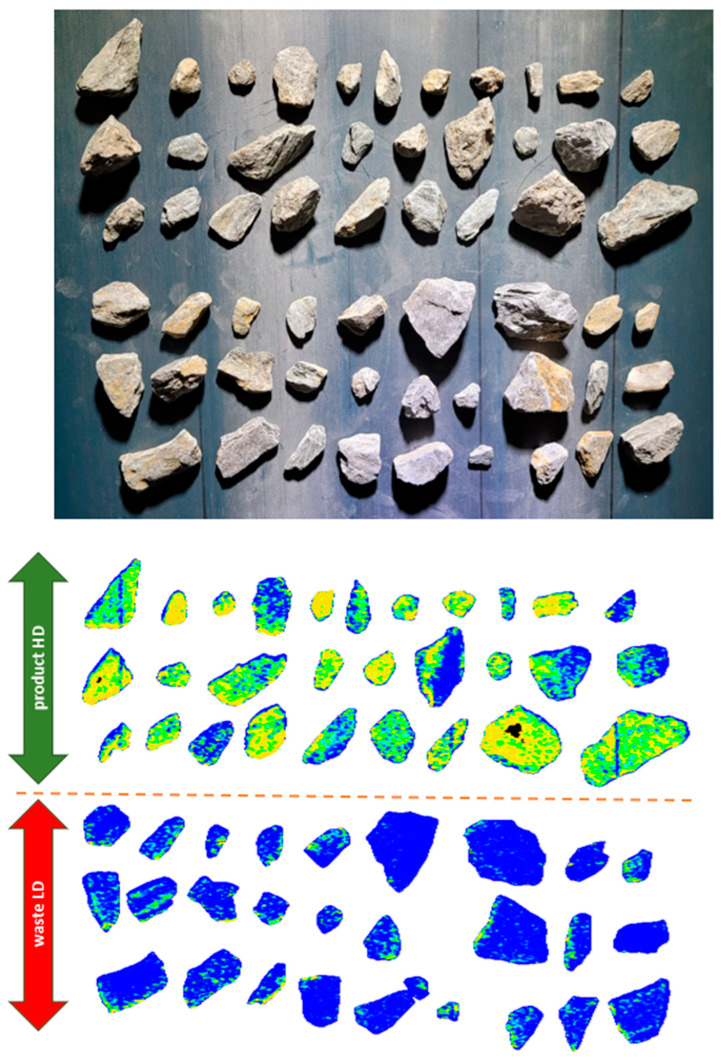
Visualization of the cut-off point for sorting results division: High-Density product and Low-Density waste.

After setting the parameters, preliminary sorting was performed to evaluate the separation efficiency—the results are presented in Figure 5, clearly showing the division between the High-Density quality class and the Low-Density quality class.

Objects containing both types of material were classified as product to avoid losing valuable components at this processing stage (perhaps for this type of grain, creating a third sorting product is recommended, with the result directed to a crushing process for a smaller grain size class to liberate the valuable component and enable the next step of pre-concentration).

### 3.3. Pre-Concentration Results

#### 3.3.1. Sorting Results

As a result of conducting the test on the prepared feed sample with the separation parameter settings, a product-to-waste mass split close to 1:1 was obtained.

**Figure 6:**
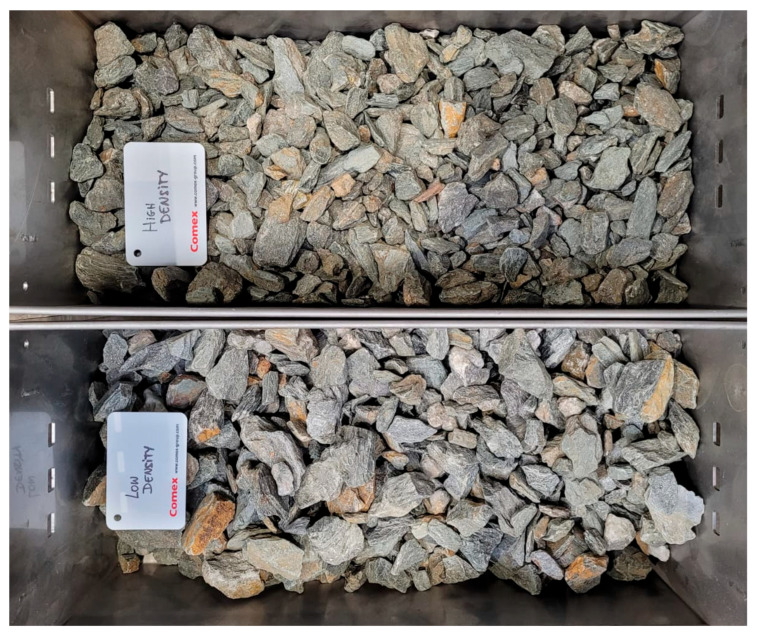
Photo of the material separated as a result of sorting: High Density at the top, Low Density at the bottom.

**Figure 7:**
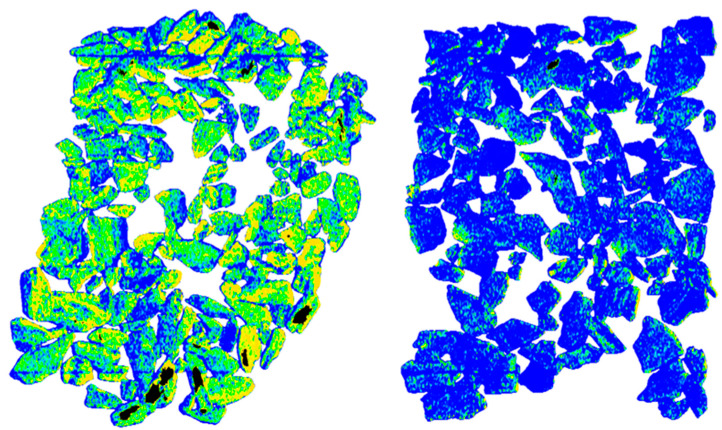
XRT scan of the sorting results confirming the correct division between product and waste: High Density on the left, Low Density on the right.

The feed preparation method had a 2:1 ore-to-waste rock ratio after selective mining, so a greater-than-initial amount of material ended up in the waste—this result is justified only if the separation outcome shows a clear division between the High-Density fraction and the Low-Density fraction (Figure 6 and Figure 7), and chemical assays additionally confirm a higher valuable component content in the pre-concentrate compared with the ore after selective mining.

#### 3.3.2. Chemical Analysis Results

Individual samples were sent for chemical analysis, which was performed using the XRF method. 

**Figure 8:**
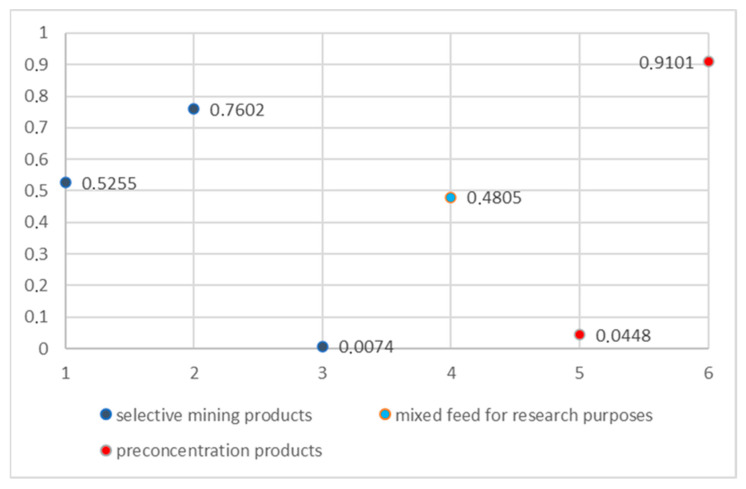
Copper content in individual samples; 1—ORE 8–16 mm, 2—ORE 16–32 mm, 3—Waste Rock 8–32 mm, 4—MIXED Feed 8–32 mm, 5—Sorting result—Waste LD, 6—Sorting result—Product HD.

The analysis results are presented in the tables (Table 5, Table 6 and Table 7) and Figure 8.

#### 3.3.3. Sorting Efficiency Results

Having the mass balance distribution of the process and measurements of the valuable component content in individual samples enables the development of a pre-concentration process flowsheet along with the calculation of the yield and the recovery rate.

The mass split between product and waste streams, combined with chemical assays of feed, product, and waste, forms the basis for constructing a detailed mass balance diagram. Recovery rate in mineral processing (Ɛ) is then computed using the standard Formula (1):

Ɛ (%) = mineral content in product/mineral content in feed × product yield × 100%(1)

**Figure 9:**
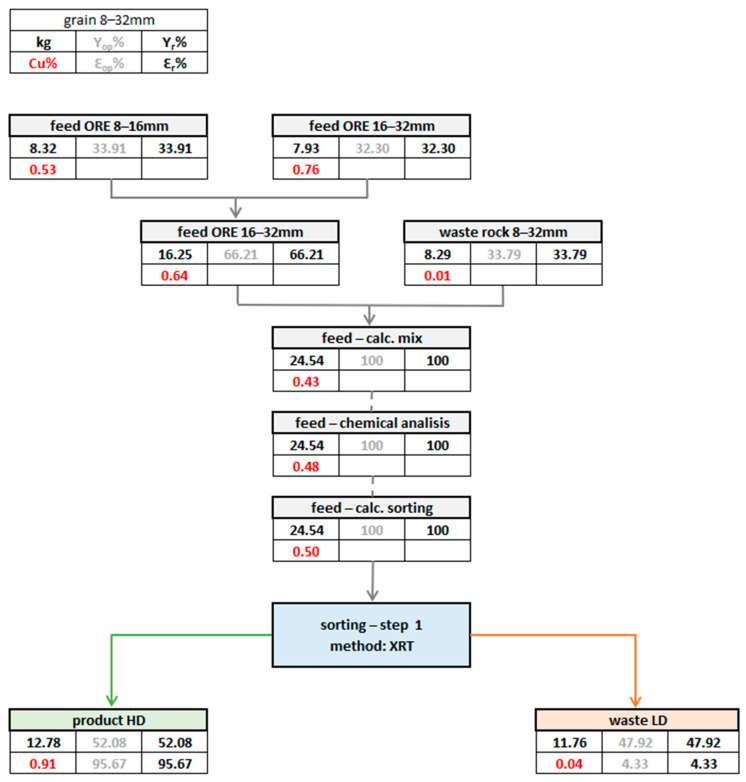
Schematic of the mass balance and chemical analysis results showing the final values of the test, including recovery parameters; Ɛ—recovery rate, Y—yield, Cu%—mineral (copper) content.

This result (Figure 9) demonstrates a 1.82x grade upgrade while rejecting nearly half the feed mass as low-value (Cu = 0.04%) waste, with minimal valuable metal loss (Ɛ < 5%). Such outcomes highlight the efficacy of density-based sorting for copper ore pre-concentration, aligning with industry benchmarks where >90% recovery at 20–40% mass pull optimizes downstream processing efficiency.

The feed for the sorting test was given in three variants:

1. According to the calculation of parameters for partial feeds before mixing.
2. According to the chemical analysis of the mixed feed.
3. According to the calculation of sorting product parameters.

The differences observed among the three copper grade values (Table 8)—(1) calculated from parameters of partial feeds before mixing, (2) from the chemical analysis of the mixed feed, (3) from sorting product parameter calculations—likely result from ore heterogeneity. When sampling small feed masses, this inherent variability can cause deviations between individual measurements.

The reported standard deviation (SD = 0.029439) and variance (0.000867) indicate low to moderate variability. This level of error is typical for small-scale sorting trials and does not invalidate the results; instead, it underscores the need for larger sample masses or replicate assays to further reduce the sampling error in future work.

## 4. Discussion

The present research results demonstrate that sensor-based pre-concentration using XRT sensors achieves a superior performance compared with traditional mining without ore grade enhancement for this specific copper ore case, delivering 95.67% Cu recovery, a 1.82x grade upgrade, and nearly 48% waste rejection.

Compared with selective mining, XRT sensor-based pre-concentration also yields the following better results:

- Selective mining produced enriched ore at 0.64% Cu with a 66.21% mass yield.
- XRT pre-concentration achieved 0.91% Cu at a 52.08% mass yield.

These findings further suggest that combining pre-concentration with selective mining, rather than using either method independently, holds greater potential for optimization. A forthcoming publication will investigate this synergy between selective mining and sensor-based sorting.

Building on these outcomes, future work will prepare sufficient material volumes for trials examining the impacts on downstream mineral processing (grinding and flotation). Subsequent iterations include the industrial-scale validation of the results, followed by comprehensive economic and environmental assessments of the proposed technology implementation.

In conclusion, this study provides a conceptual foundation for a systematic investigation into the integrated application of selective mining and sensor-based ore sorting. By establishing a direct comparison between the two approaches, we have outlined a framework for assessing their individual and combined contributions to overall process optimization. Recognizing the complexity of industrial ore variability and the importance of representative sampling, future papers in this series will address the performance of sensor-based sorting using XRT, XRF, and SWIR analysis on intermediate ore grades, heterogeneous feed materials, and real-life mining scenarios with varying degrees of selectivity. Through this planned sequence of studies, we aim to develop a comprehensive understanding of how selective mining and pre-concentration can be jointly optimized to enhance resource efficiency, improve subsequent mineral processing stages (milling and flotation), and maximize the economic value of copper mining operations.

## Figures and Tables

**Table 1:** Content of copper in feed sample “ORE 8–16 mm”.

Value	Cu [%]
Measurement 1	0.519500
Measurement 2	0.527200
Measurement 3	0.529800
Mean measurement	0.525500
Standard deviation	0.004373
Variance	0.000019

**Table 2:** Content of copper in feed sample “ORE 16–32 mm”.

Value	Cu [%]
Measurement 1	0.759400
Measurement 2	0.739900
Measurement 3	0.781200
Mean measurement	0.760167
Standard deviation	0.016869
Variance	0.000285

**Table 3:** Content of copper in feed sample “Waste Rock 8–32 mm”.

Value	Cu [%]
Measurement 1	0.008220
Measurement 2	0.007260
Measurement 3	0.006800
Mean measurement	0.007427
Standard deviation	0.000592
Variance	0.000000

**Table 4:** Content of copper in feed sample “MIXED Feed 8–32 mm”.

Value	Cu [%]
Measurement 1	0.494400
Measurement 2	0.469800
Measurement 3	0.477400
Mean measurement	0.480533
Standard deviation	0.010284
Variance	0.000106

**Table 5:** Content of copper in sample “Sorting result—Waste Low Density”.

Value	Cu [%]
Measurement 1	0.045810
Measurement 2	0.045130
Measurement 3	0.043340
Mean measurement	0.044760
Standard deviation	0.001042
Variance	0.000001

**Table 6:** Content of copper in sample “Sorting result—Product High Density”.

Value	Cu [%]
Measurement 1	0.905200
Measurement 2	0.915800
Measurement 3	0.909300
Mean measurement	0.910100
Standard deviation	0.004364
Variance	0.000019

**Table 7:** Summary of mean measurements for copper content in the analyzed samples.

Sample	Cu [%]
“ORE 8–16 mm”	0.525500
“ORE 16–32 mm”	0.760167
“Waste Rock 8–32 mm”	0.007427
“MIXED Feed 8–32 mm”	0.480533
“Sorting result—Waste LD”	0.044760
“Sorting result—Product HD”	0.910100

**Table 8:** Content of copper in feed.

Feed According to	Cu [%]
The calculation of parameters for partial feeds before mixing	0.430000
The chemical analysis of the mixed feed	0.480000
The calculation of sorting product parameters	0.500000
Mean measurement	0.470000
Standard deviation	0.029439
Variance	0.000867

## Data Availability

The original contributions presented in this study are included in the article. Further inquiries can be directed to the corresponding author.
